# Genetic Mapping of Climbing and Mimicry: Two Behavioral Traits Degraded During Silkworm Domestication

**DOI:** 10.3389/fgene.2020.566961

**Published:** 2020-12-17

**Authors:** Man Wang, Yongjian Lin, Shiyi Zhou, Yong Cui, Qili Feng, Wei Yan, Hui Xiang

**Affiliations:** ^1^Guangdong Provincial Key Laboratory of Insect Developmental Biology and Applied Technology, Institute of Insect Science and Technology, School of Life Sciences, South China Normal University, Guangzhou, China; ^2^Guangdong Provincial Key Laboratory of Biotechnology for Plant Development, School of Life Sciences, South China Normal University, Guangzhou, China

**Keywords:** *Bombyx mori*, behavioral domestication, climbing, mimicry, bulked segregant analysis, selection sweep screening

## Abstract

Behavioral changes caused by domestication in animals are an important issue in evolutionary biology. The silkworm, *Bombyx mori*, is an ideal fully domesticated insect model for studying both convergent domestication and behavior evolution. We explored the genetic basis of climbing for foraging and mimicry, two degraded behaviors during silkworm domestication, in combination of bulked segregant analysis (BSA) and selection sweep screening. One candidate gene, *ASNA1*, located in the 3–5 Mb on chromosome 19, harboring a specific non-synonymous mutation in domestic silkworm, might be involved in climbing ability. This mutation was under positive selection in Lepidoptera, strongly suggesting its potential function in silkworm domestication. Nine candidate domesticated genes related to mimicry were identified on chromosomes 13, 21, and 27. Most of the candidate domesticated genes were generally expressed at higher levels in the brain of the wild silkworm. This study provides valuable information for deciphering the molecular basis of behavioral changes associated with silkworm domestication.

## Introduction

Compared with wild animals, domestic animals showed typical behavioral adaptation to artificial selection. Reduced fear of humans and increased tolerance to artificial stresses are two earliest and most important adaptations in animals. Artificial selection can reduce the effects of natural selection of animals, for example, foraging ability and predator avoidance (Solberg et al., 2020). Domesticated behaviors shared in disparate animals are considered as “domestication syndrome” (Wilkins et al., 2014). Deciphering the genetic basis of domesticated behaviors will improve the understanding of animal domestication. The developments of high-throughput sequencing and evolutionary genomics have greatly accelerated the reverse genetic studies on morphological traits in mammals and poultries (Frantz et al., 2015; Qiu et al., 2015; Pendleton et al., 2018; Zhang et al., 2018). However, behavioral traits are more difficult to assay than morphological traits in animals. Mapping behavior-related loci in animals is largely hampered by the difficulties in constructing mapping populations and phenotype investigation.

During the breeding of silkworm, many behavioral and morphological traits have been domesticated. Domesticated genes and pathways involved in the nervous system in silkworm were also found in other animals, suggesting the “domestication syndrome” in silkworm (Xiang et al., 2018). The larval locomotion, especially the climbing for foraging ability, has been largely decreased in the domestic silkworm. Notably, the domestic silkworm has the greatest potential to climb at the wandering stage or been infected by baculovirus, indicating possible correlations with the nervous system (Kamita et al., 2005; Feiguin et al., 2009). Another novel adapted behavior in the domestic silkworm is the loss of larval mimicry response to exogenous stimulus. Usually, the wild silkworm larva at the end of instar stretches its body and holds tightly to imitate a mulberry branch immediately in response to exogenous stimulus. The climbing and mimicry abilities are essential for foraging and predator avoidance in the wild silkworm and other wild insects (Garrouste et al., 2016). However, the genetic basis for these two interesting behavioral traits is still unclear. Benefit from the high-throughput sequencing-based bulked segregant analysis (BSA), the trait-related loci can be identified rapidly using individuals with extreme phenotypes in a mapping population (Takagi et al., 2013; Zegeye et al., 2018; Liu et al., 2019). The feasible construction of mapping population between the wild silkworm and the domestic silkworm also enables the identification of domestic behavior-related loci in silkworm.

In this study, the two domestic behaviors, climbing for foraging and mimicry, were carefully observed in the mapping population between the wild silkworm and the domestic silkworm. Individuals showing fully loss of climbing ability for foraging or mimicry response were selected for bulked re-sequencing and compared with both parents and individuals with extreme climbing ability and mimicry response. Candidate genes related to these two behaviors were identified through BSA analysis, combined with selection sweep screening and estimation of expression patterns of domesticated genes in candidate region(s). This study provides an efficient method to illustrate the potential genetic basis related to behavioral evolution during silkworm domestication. Further study on behavior-related genes will provide important clues for understanding the genomic evolution of behavioral adaptation under artificial selection in animals.

## Materials and Methods

### Construction of Mapping Population

The wild silkworm with climbing ability and mimicry response and the domestic silkworm P50 strain loss of these two characters were collected from a wild field in Zhejiang Province and maintained as indoor populations. The wild female moth with larger body and weaker flying ability was crossed with the domestic moth P50 (male) to generate the F1 generation. Then, the F1 (male) was backcrossed with the domestic moth P50 strain (female) to generate the BC1 mapping population. Climbing and mimicry behaviors were carefully observed in both F1 and BC1 generations. All the silkworms were fed at 27°C and 70% humidity.

### Phenotypic Assay and Establishment of Segregation Groups

To examine the climbing ability, the BC1 individuals (∼200) were fed in boxes (with insufficient mulberry leaves) under mulberry seedlings at the beginning of the 5th instar for ∼7 days. Individuals with strong climbing ability could climb up ∼50 cm to the mulberry seedlings for foraging. Those climbed up to the branches were put back to the box for repeated observation (at least 3 times/day). Individuals that consistently climb or stay in boxes were considered as individuals with extreme distinct phenotypes in the segregation population and selected for bulked sequencing. We also observed individuals with white or dark body in both segregation groups. Therefore, four independent sets of different larval body colors were generated for re-sequencing, i.e., white body with climbing ability, white body loss of climbing ability, dark body with climbing ability, and dark body loss of climbing ability, 20 individuals for each set.

To estimate the mimicry traits, the BC1 individuals (∼200) were fed on mulberry branches. Then, the branches were shaken artificially to observe the mimicry response. Those extended their head and chest to simulate the dendrite shape and maintained for at least 10 s were considered as extreme mimic individuals (Supplementary Video 1). Those showed mimicry response were also verified by hand touch (Supplementary Video 2). On the contrary, individuals that kept eating even when touched by hand were considered as extreme non-mimic individuals (Supplementary Video 3). Eventually, 18 individuals with strong mimicry response and 20 non-mimicry individuals were selected for re-sequencing, respectively.

### DNA Extraction and Sequencing

The total DNA was extracted for each individual using a traditional phenol–chloroform DNA extraction protocol and quantified by NanoDrop 2000 (Thermo). Equal quantity (1.5 μg) DNA of each individual in each set was pooled to obtain bulked DNA for sequencing on the Illumina Hiseq 4000 platform (PE150, with insert size around 350 bp). The sequencing libraries were constructed according to the manufacturer’s instructions. In addition, both the wild silkworm (P_Wild) and the domestic silkworm (P_P50) were sequenced for comparison.

### Sequence Alignment and SNP/InDel Calling

The reference genome of domestic silkworm was downloaded from the Silkbase^1^. The re-sequencing short reads of each bulk were aligned to the reference genome with BWA (Burrows–Wheeler Aligner) (-t 4 -k 32 -M -R) (Li and Durbin, 2009). Alignment files were converted to BAM files using the SAMtools software (-bS -t) (Li et al., 2009). Potential PCR duplications were excluded using the rmdup in SAMtools. If multiple read pairs were mapped to identical coordinates, only the pair with the highest mapping quality was retained. Reliable genome-wide single-nucleotide polymorphisms (SNPs) and insertions/deletions (InDels) were identified with the Unified Genotyper function in GATK (–filterExpression “QD < 4.0 ∥ FS > 60.0 ∥ MQ < 40.0,” -G_filter “GQ < 20,” –cluster WindowSize 4) (McKenna et al., 2010). InDels were further filtered with the Variant Filtration parameter (–filter Expression “QD < 4.0 ∥ FS > 200.0 ∥ Read PosRankSum < −20.0 ∥ Inbreeding Coeff < −0.8 ”). ANNOVAR was used to annotate SNP or InDel based on the GFF3 files of the reference genome (Wang et al., 2010). The gene models were derived from Xiang et al. (2018) that integrative assembled with GLEAN based on a combination of coding sequences from SilkDB, homology, and *ab initio* sets (AUGUSTUS, SNAP, and GENSCAN).

### SNP/InDel Index Calculation

The homozygous SNPs/InDels between the two parents were extracted as markers for index calculation. Only SNPs/InDels covered by at least seven reads in both parents (∼1/3 of the sequencing depth of parents) were considered. The genotype of the P50 strain was used as the reference, and reads supporting the reference genotype or non-reference genotype were counted in each bulk. The SNP index and InDel index were calculated with reads supporting the non-reference genotype divided by the total reads in each sequenced bulk (Takagi et al., 2013). SNPs/InDels with depth < 7 in both segregation groups or missing in one group were excluded. The genome-wide SNP/InDel indexes were visualized in sliding window of 1 Mb with a step size of 10 kb. The average index of all the SNPs/InDels presented in each window was designated as the index of this window in each group. The ΔSNP/InDel index was calculated to represent the difference of index between the segregation groups, and 1,000 permutation tests were performed with 95% confidence level as threshold. The SNP/InDel index in each segregation group and the ΔSNP/InDel index were shown with ggplot package in RStudio.

### Screening of Domestication Signature

Genes related to domesticated behaviors are usually under selection. Based on the published genome-wide SNPs of 137 domestic silkworm and 7 wild silkworm strains (Xiang et al., 2018), we calculated the selection signatures (*Fst* and π) of the chromosomes harboring the candidate regions related to climbing and mimicry to screen candidate domesticated genes as described previously (Zhu et al., 2019a). The allele frequency for each site was calculated with the reads supporting the reference genotype divided by the total covered reads. The effects of candidate SNPs were then estimated according to the annotation of the reference genome.

### Evolution Analysis of the Candidate Genes Related to Climbing in Lepidoptera

The homologous sequences of candidate genes related to climbing were obtained using blastp against the NCBI database non-redundant nucleotide library^2^ and the sequences of *Antheraea yamamai* in the GigaDB database^3^ (Kim et al., 2018). Protein sequences with at least 80% identity in other insect genomes were downloaded for comparisons, including the five Lepidoptera species *A. yamamai*, *Danaus plexippus*, *Chilo suppressalis*, *Helicoverpa armigera*, and *Papilio machaon*. The downloaded protein sequences were aligned with ClustalW using default settings in MEGA 7 (Kumar et al., 2016). The minimum-evolution tree was constructed to show the evolution of candidate genes in all the collected insect genomes. Branch model (model = 0 and NSsites = 0 for one-ratio model, model = 2 and NSsites = 2 for two-ratio model) and branch-site model (model = 2, NSsites = 2) in PAML software (version 4.8) were used for phylogenetic analysis (Yang, 2007). The likelihood ratio test was used to estimate the fitness of different models.

### Transcriptomic Analysis

RNA-seq data from different tissues of *B. mori* (5th instar day 3 larvae) were downloaded from the NCBI SRA database^4^. The accession numbers are SRR4425245 (ovary), SRR4425244 (testis), SRR4425250 (brain), SRR4425254 (anterior silk gland), SRR4425258 (middle silk gland), SRR4425260 (posterior silk gland), DRR095110 (midgut), SRR4425248 (fat body), SRR7812745 (integument), and DRR095113 (malpighian tubule). Reads were mapped to the silkworm reference genome with TopHat2 (Kim et al., 2013), and the expression levels (FPKM) were further determined with Resm (Li and Dewey, 2011). The expression heatmaps were constructed with normalized Z-scores on the OmicShare online platform^5^.

We also sequenced the RNA of the brains of both parents at three key larval stages (middle larval stage, late larval stage, and wandering stage) to estimate the expression levels of candidate genes related to climbing and mimicry, three replicates for each sample. Reads were mapped to the reference genome of *B. mori* with TopHat2 (Kim et al., 2013). The expression levels (FPKM) of genes were further calculated with Cuffdiff (Trapnell et al., 2012). The expression heatmaps were constructed as described above. We also normalized the FPKM of each candidate gene according to the total numbers of sequencing depth of the corresponding samples, and Student’s *t*-test was imported for estimation of the expression differences between wild silkworm and domestic silkworm.

## Results

### Inheritance of Climbing and Mimicry Behaviors

**FIGURE 1:**
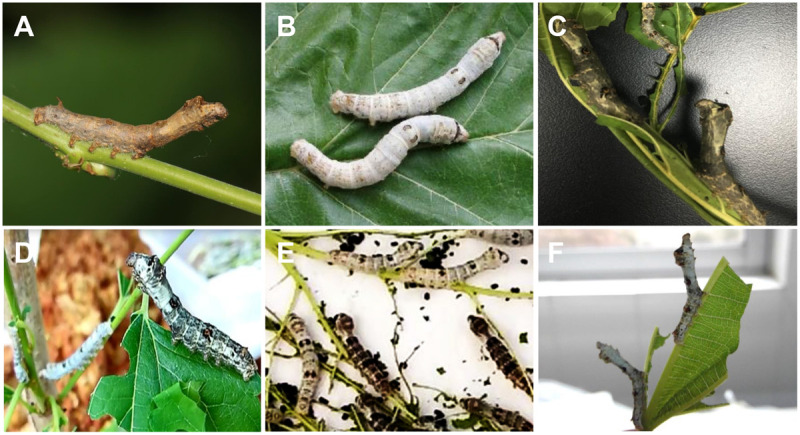
Larvae of the wild silkworm, the domestic silkworms, and the hybrid populations. **(A)** The wild silkworm with the ability of climbing and mimicry. **(B)** The domestic silkworm unable to climb for foraging and mimicry. **(C)** F1 hybrids of the wild silkworm and the domestic silkworm. **(D)** Individuals with climbing ability. **(E)** Individuals unable to climb. Individuals with white or dark body color are observed in both the two segregation groups. **(F)** Backcrossed individuals with typical mimicry response.

The wild female moth with larger body and weaker flying ability was crossed with the domestic silkworm P50 strain (male) to generate the F1 generation (Figures 1A–C). Interestingly, all of the individuals in the F1 generation showed a strong ability of climbing and mimicry response, indicating that these two behaviors were dominant. The F1 was then backcrossed with the recessive parent (the domestic silkworm P50 strain) to generate the BC1 populations. Both climbing for foraging and mimicry response were segregated in the BC1 populations (Supplementary Videos 1–3). Individuals in BC1 with stronger climbing ability could climb up to over 50 cm from the foot of the mulberry seedling. We also found that the climbing ability for foraging was not linked to the body color (Figures 1D,E), i.e., individuals with strong climbing ability were detected in both individuals of white and dark bodies. Individuals in BC1 with strong ability to mimic could response to artificial shake and hand touch promptly and maintain for at least 10 s (Figure 1F and Supplementary Videos 1, 2). Those unable to mimic showed no response to artificial shaking and hand touching (Supplementary Video 3).

### BSA to Identify the Candidate Region Related to Climbing Behavior

Because the body color was not linked to the climbing for foraging behavior, we therefore generated four bulks to identify the candidate region related to climbing, i.e., white body with climbing ability, dark body with climbing ability, white body unable to climb, and dark body unable to climb (Figures 1D,E and Supplementary Table S1). In total, 14.7–18.2 Gb data were obtained for the four bulks (Supplementary Table S1). The clean reads were mapped to the reference genome for SNP and InDel calling as described in “Materials and Methods” section. More than 93.34% clean reads could be aligned properly, covering 28–34 × of the reference genome in depth (Supplementary Table S1). The two parents, the wild silkworm (P_Wild) and the domestic silkworm P50 (P_P50), were also sequenced to 10.9 Gb (20.39 × in depth) and 10.0 Gb (21.08 × in depth) for comparison, respectively (Supplementary Table S1). Only SNPs/InDels with homozygous genotypes in both parents were extracted as markers for further analyses, resulting in 7,142,097 SNPs and 346,910 InDels in the white set and 7,063,453 SNPs and 340,067 InDels in the dark set, respectively.

To identify the candidate loci related to climbing, the genotypes of the parent P_P50 were used as reference for calculating the SNP/InDel index in sequenced bulks. If all short reads covering one position are identical to the genotype of P_P50, the SNP/InDel index will be 0. In contrast, if all of the short reads support a different genotype from the reference, the SNP/InDel index will be 1. The ΔSNP/InDel index was also calculated to represent the index differences between the climbing ability bulks and the non-climbing ability bulks. Ideally, the candidate loci related to climbing behavior are expected to be heterozygous, and the ΔSNP/InDel index should be 0.5. The candidate region will be outstanding in the ΔSNP/InDel index due to the strong linkages between closely linked SNPs/InDels with the causal mutation.

**FIGURE 2:**
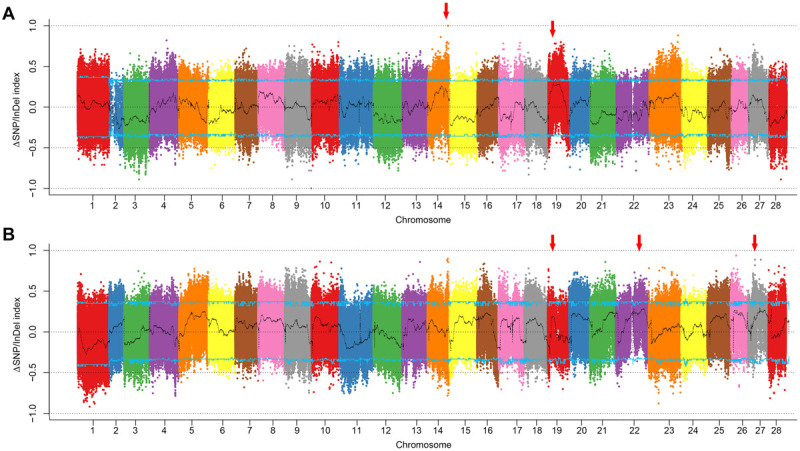
Plotting of ΔSNP/InDel index of the climbing and non-climbing bulks. **(A)** White body color set. **(B)** Dark body color set. Curves in black are indexes calculated with 1 Mb sliding window with 10 kb step size. Blue lines indicate the cutoff of 95% confidence interval. Red arrows indicate the candidate regions for climbing.

**FIGURE 3:**
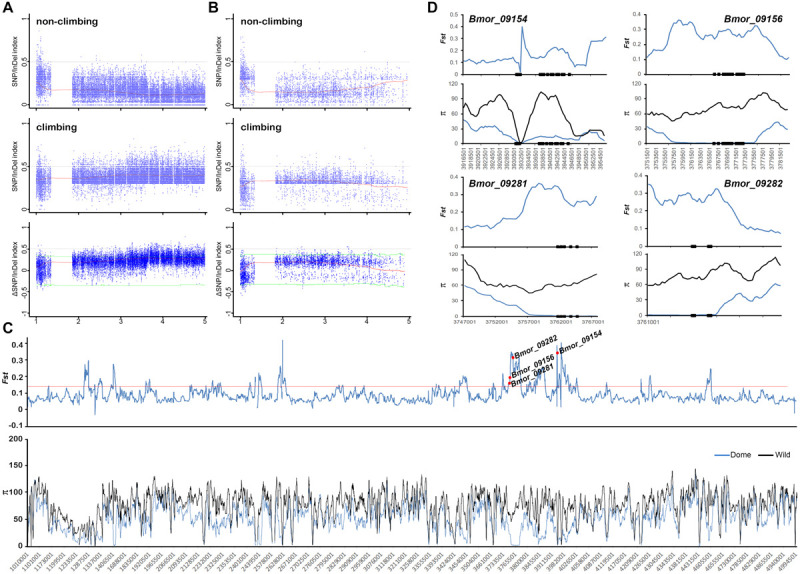
Mapping of climbing-related loci through BSA analysis combined with selective sweep approaches. SNP/InDel index information of the candidate region (1–5 Mb on chromosome 19) detected by BSA in both the white color set **(A)** and the dark color set **(B)**. The first panel is the index in the group with climbing ability, and the second panel is the index in the group loss of climbing ability. The ΔSNP/InDel index shown in the third panel represents the difference of index between these two groups. Red curves indicate the index calculated with 1 Mb sliding window with 10 kb step size. Green lines indicate the cutoff of 95% confidence interval. **(C)** Selective sweep screening in the candidate region. Signature index, including population divergence coefficient (*Fst*) between the early domestic silkworm group and the wild silkworm group, and nucleic acid diversity (π) in the silkworm population are shown along the chromosome. Red line represents the top 5% *Fst* cutoff. The four domesticated genes are marked with red circles. Dome, domestic silkworm; Wild, wild silkworm. **(D)** Selection signatures of the four candidate domestication genes. The black blocks represent the genomic structure of corresponding genes.

In comparison of the climbing and non-climbing bulks of white body, two candidate peaks located on chromosomes 14 and 19 were identified (Figure 2A, Supplementary Figure S1, and Supplementary Data). The peak on chromosome 19 nearly reached the 95% confidence interval. Interestingly, this peak was also detected in the comparison of climbing and non-climbing bulks of dark body (Figure 2B, Supplementary Figure S2, and Supplementary Data), indicating a strong linkage to the climbing behavior. By overlapping the SNPs/InDels with high index in both sets, the candidate region could be roughly narrowed down to 3–5 Mb interval on chromosome 19 (Figures 3A,B).

### Identification of Candidate Domesticated Genes Related to Climbing by Selective Sweep Screening

**FIGURE 4:**
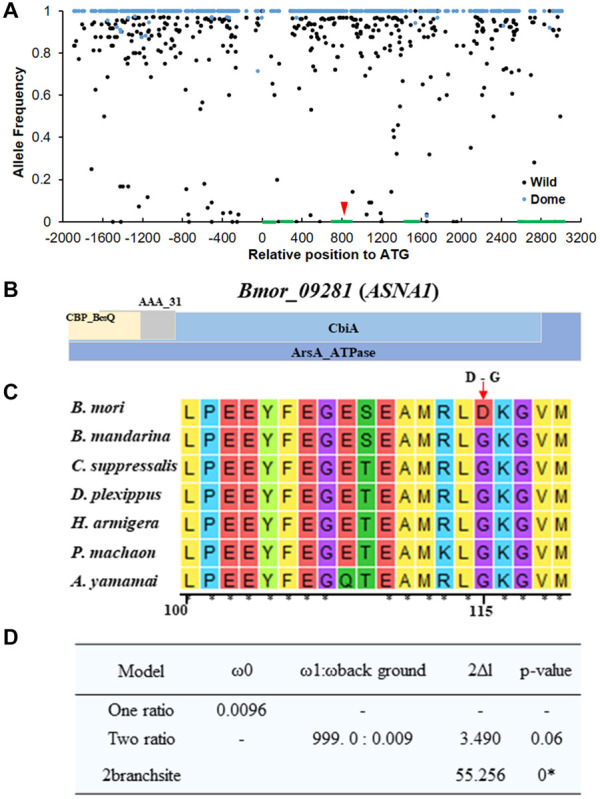
SNP analysis of the candidate domesticated gene *Bmor_09281* (*ASNA1*) related to climbing ability. **(A)** Allele frequencies in the genetic region of *Bmor_09281* (*ASNA1*). Green blocks indicate the genomic structure of the genes. Red arrow shows the non-synonymous replacement in the domestic silkworm group compared with the wild silkworm group. Black dots and blue dots represent the allele frequencies in wild silkworm and domestic silkworm, respectively. **(B)** Predicted conserved domains in *ASNA1*. **(C)** Protein alignment of *ASNA1* with homologous collected from six other Lepidoptera species. The functional domain of *ASNA1* is shown in the top panel. Red arrow shows the non-synonymous replacement specific in the domestic silkworm. **(D)** Evolution analysis of *ASNA1* with PAML analysis. * significant fitness of the alternative model compared with the one ratio model using the likelihood ratio test.

Considering the possible sequencing bias and errors that occurred in the candidate region, we extended the candidate region on 1–5 Mb on chromosome 19 for the identification of candidate domesticated genes related to climbing. We screened the selective sweep of domestication signatures in this region to discover candidate domesticated genes as described in “Materials and Methods” section (Figure 3C). As expected, strong selective sweep signatures were found in this region, especially in 3.7–4 Mb, in which four candidate domesticated genes were identified (Figures 3A–C). These four candidate domesticated genes were annotated as fanconi-associated nuclease 1-like (*FAN1*), GPI ethanolamine phosphate transferase 3 (*Pigo*), ATPase ASNA1 homolog (*ASNA1*), and uncharacterized protein, respectively (Supplementary Table S2). These four genes showed remarkable higher *Fst* and extremely lower π in the domestic silkworm than in the wild silkworm (Figures 3C,D). In the Silkworm Genome Informatics Database (SGID) (Zhu et al., 2019b), *FAN1*, *Pigo*, and *ASNA1* were possibly under positive selection (Supplementary Table S2). Consistently, the allele frequencies in this region were also quite different between the domestic silkworm and the wild silkworm, with nearly fixed alleles in the domestic silkworm population (Figure 4A and Supplementary Figure S3). Interestingly, non-synonymous variations divergent in allele frequency between the domestic and the wild silkworm were identified in the coding regions of *FAN1*, *Pigo*, and *ASNA1* (Figure 4A and Supplementary Figure S3). To further explore the potential significance of these non-synonymous variations, the homologous protein sequences in 5 Lepidoptera species and 62 other insect species were collected for comparative analysis. We found that only the non-synonymous variation on the ATPase ASNA1 was specific in the domestic silkworm, causing glycine (G) to aspartic acid (D) conversion at residue 115 (Figures 4B,C and Supplementary Figures S4, S5). This variation also appeared to be positively selected in the domestic silkworm clade, as detected in PAML analyses (*p* < 0.0001 using the branch-site model and *p* = 0.06 using the branch model) (Figure 4D). Of the 68 collected species, *ASNA1* was a single copy gene in *B. mori* and *B. mandarina* and possibly two copies in only four species (Supplementary Figure S4). The amino acids affected by the non-synonymous variations identified on FAN1 (G_10__5_D and D_15__5_E) and Pigo (K_10__8_E and I_46__3_V) were common between *B. mori*, *B. mandarina*, and some of the other Lepidoptera species, suggesting that these variations were not correlated with the loss of climbing ability in domestic silkworm (Supplementary Figure S6).

### Mapping of Mimicry-Related Loci by BSA Analysis and Selective Sweep Screening

**FIGURE 5:**
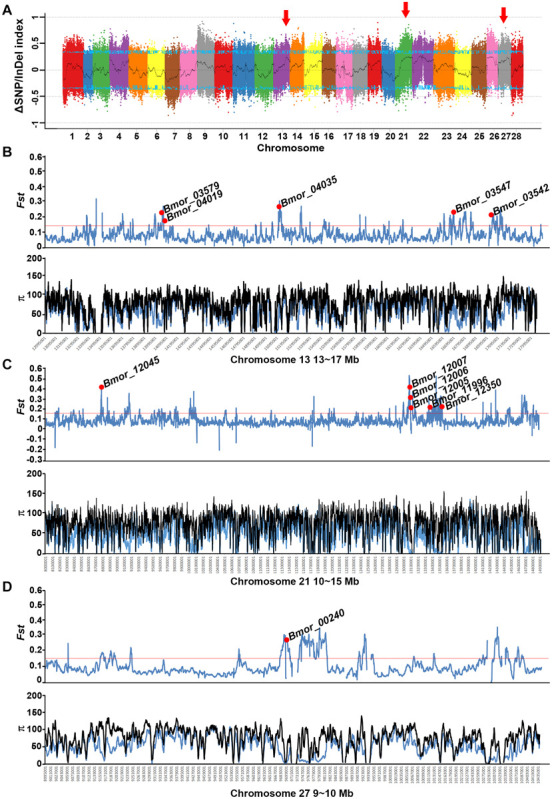
Preliminary mapping of mimicry-related loci through BSA analysis combined with selective sweep approaches. **(A)** ΔSNP/InDel index of the mimicry and non-mimicry bulks. The candidate regions related to mimicry are marked with red arrows. **(B–D)** The selective sweep signatures in the three potential candidate regions located on chromosomes 13, 21, and 27. Black curves indicate the index calculated at 1 Mb sliding windows scale with 10 kb step size. Blue lines indicate the cutoff of 95% confidence interval. Red lines indicates 5% *Fst cutoff*.

To identify candidate regions related to the mimicry phenotype, individuals with/without responses to artificial shaking or hand touching were collected and pooled separately for re-sequencing. The clean data covered about 32- and 37-fold of the reference genome in depth for mimicry and non-mimicry bulks, respectively (Supplementary Table S1). Compared with the genotypes of the recessive parent P_P50, there were 1,877,029 SNPs and 333,064 InDels retained for BSA. Candidate peaks related to the mimicry trait were detected in 13-17 Mb on chromosome 13, 10–14 Mb on chromosome 21, and 9–10 Mb on chromosome 27, respectively (Figure 5 and Supplementary Figure S7). However, all the peaks were not beyond the 95% confidence interval, suggesting the difficulty of accurate mapping of behavior traits by BSA. Screening of the selective signatures resulted in 12 genes in total in these regions (Figures 5B–D and Supplementary Table S2). These genes were predicted to encode proteins with diverse functions, such as neuropeptide receptors, glycine receptor subunit alpha-4, transporters, kinase related genes, and TBC family member (Supplementary Table S2). Of these candidate genes, only six genes were predicted to be under positive selection in silkworm in SGID (Supplementary Table S2). The allele frequencies in these candidate genes were much higher in domestic silkworm than in wild silkworm (Supplementary Figure S8).

### Tissue Expression Pattern of the Candidate Domestication Genes Potentially Related to Climbing and Mimicry

**FIGURE 6:**
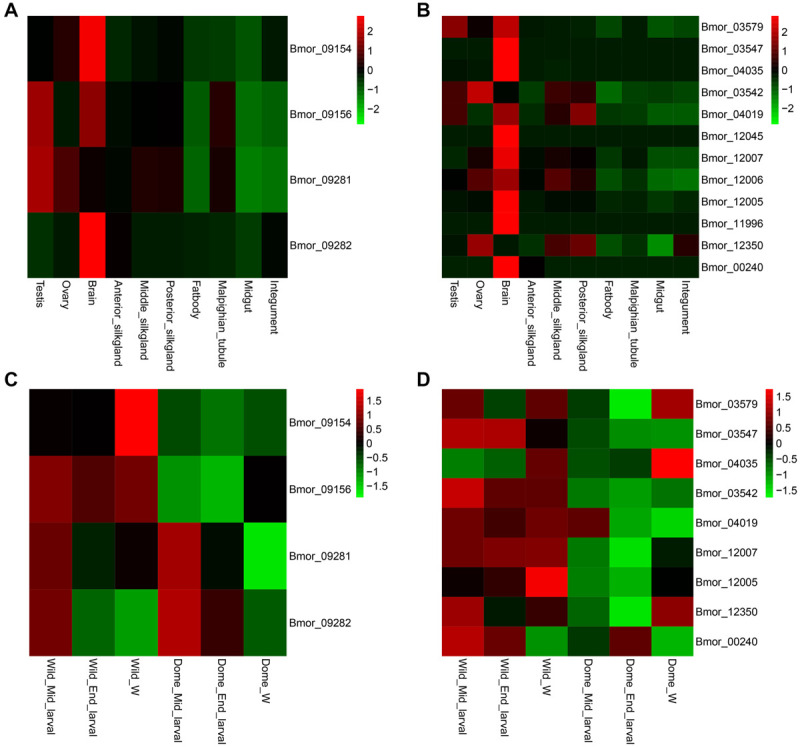
Expression patterns of the candidate genes related to climbing and mimicry. Tissue expression pattern of the candidate domesticated genes related to climbing **(A)** and mimicry **(B)**. Expression levels of the candidate domesticated genes related to climbing **(C)** and mimicry **(D)** at different larval stages of the wild silkworm and domestic silkworm. Dome, domestic silkworm; Wild, wild silkworm; End_larval, the end of the last larval stage; Mid_larval, the middle of the last larval stage; W, wandering stage.

By investigating the published RNA-seq data of the domestic silkworm, we noticed that most of the candidate genes related to climbing for foraging and mimicry were expressed at higher levels in the brain than in the other tissues (Figures 6A,B), especially the mimicry-related candidate genes (Figure 6B). These patterns implied that the loci related to domesticated behaviors might primarily function in the brain. Therefore, we further compared the expression of these genes in the brain of the wild and domestic silkworms at three key larval developmental stages (middle larval stage, late larval stage, and wandering stage) as described in “Materials and Methods” section (Figures 6C,D). The expressions of *FAN1* and *Pigo* were higher in the wild silkworm than in the domestic silkworm but no significant differences in *ASNA1* (*p* = 0.2820 for middle larval stage brain, *p* = 0.2730 for late larval stage brain, and *p* = 0.1917 for wandering stage brain, respectively) (Figure 6C and Supplementary Figure S9). We also verified the expression levels of candidate genes related to mimicry in the brains of wild and domestic silkworms. It is interesting that most candidate genes related to mimicry except the three non-detected genes were generally expressed at higher levels in the brain of the wild silkworm (Figure 6D and Supplementary Figure S9).

## Discussion

In this study, we provided a novel example of behavioral domestication in silkworm. Degraded climbing for foraging and loss of mimicry in silkworm are attributed to tolerance to artificial condition and relaxation of natural selection. These behavior traits are usually designated as “domestication syndrome” (Wilkins et al., 2014). Particularly, mimicry is a special adaptive behavior formed over long-term evolution of insects in nature. We had observed that the larval wild silkworms occasionally mimicked the branch, by stretching their thorax and head and remaining still (Figure 1A).

Body color is another well documented morphological trait in “domestication syndrome” (Jensen, 2014). Associations between genes controlling pigmentation with behavior features have been reported in several domestic animals (Jensen, 2014). In the dog breed German shepherd, variation in attention and activity was reported to be associated with a polymorphism of tyrosine hydroxylase (*TH*), a key enzyme in the melanin synthesis pathway (Kubinyi et al., 2012). However, we found that the body color was not correlated to the climbing for foraging in the BC1 population. Therefore, two bulks with extreme phenotypes were generated for mapping the climbing behavior for both individuals with white and dark bodies. Comparisons between the candidate regions identified in bulks with different body colors efficiently narrowed the candidate region to chromosome 19.

Behavior is controlled by the interactions among neurons in their nervous systems (Haynes, 1988; Comer and Robertson, 2001). Genome-wide investigations consistently identified candidate domestication genes in the nervous systems that might be related to domestic behaviors (Schubert et al., 2014; Dong et al., 2015; Frantz et al., 2015; Qiu et al., 2015; Lawal et al., 2018; Pendleton et al., 2018; Xiang et al., 2018). Disability of climbing for foraging in the domestic silkworm might be attributed to the changes of the nervous system. Of the four candidate domesticated genes related to climbing loci that were identified (Supplementary Table S2), *FAN1* was reported to be associated with neurological disorders, such as schizophrenia (Li et al., 2012; Zhao et al., 2014; Segui et al., 2015). *FAN1* encodes a DNA repair nuclease that inhibits the progression of DNA replication forks and prevents chromosomal abnormalities, with endonuclease and exonuclease functions (Pizzolato et al., 2015; Lachaud et al., 2016). Variation of *FAN1* can lead to a series of psychiatric and neurodevelopmental phenotypes (Ionita-Laza et al., 2014). Glycosylphosphatidylinositol (GPI) ethanolamine phosphate transferase (*Pigo*) participates in glycosylphosphatidylinositol biosynthesis. Mutation of genes involved in GPI biosynthesis can induce hyperphosphatasia with mental retardation syndrome (Krawitz et al., 2012; Chiyonobu et al., 2014; Nakamura et al., 2014; Xue et al., 2016). *Pigo* encodes GPI ethanolamine phosphate transferase 3, catalyzing the final step of GPI-anchor synthesis. Mutations in *Pigo* caused epileptic encephalopathy and led to severe neurological impairment, dysmorphia, chorea, seizures, and early death (Freeze et al., 2012; Zehavi et al., 2017). *ASNA1* encodes an ATPase targeting tail-anchored protein that is versatile and important in various biological processes. In humans, mutations in *ASNA1* caused rapidly progressive pediatric cardiomyopathy (Verhagen et al., 2019) and might be related to early onset Parkinson’s disease (Kun-Rodrigues et al., 2015). In comparisons with other Lepidoptera species, we found a non-synonymous variation specific to the domestic silkworm. This variation might be involved in the degraded climbing ability in the domestic silkworm. Although the function or biological significance of the four genes is still unknown in insects, evidence from mammals and particularly humans shed light on their roles in neural pathways. These genes were primarily expressed in the brain of the domestic silkworm. Functional verification in an insect model will be of great value.

The BSA-seq approaches in this study detected many relative weak peaks related to mimicry. Combination with selective sweep screening efficiently narrowed down the candidates. As expected, many candidate genes related to mimicry were also involved in neural pathways. For example, neuropeptide receptors were reported to be facilitators of animal domestication (Herbeck and Gulevich, 2019). Glycine receptor subunit alpha-4 highly expressed in neural tissues might function in the regulation of neuronal activity in vertebrates (Wang and Slaughter, 2005). TBC family proteins are associated with the formation of multiple functional cilia and the growth of synapses in humans (Shi et al., 2018). In flies, pathogenic mutations of TBCs caused severe neurological defects (Fischer et al., 2016). These candidate genes were mostly highly expressed in the silkworm brain and higher expressed in the brain of the wild silkworm, suggesting the importance of neural pathways in larval mimicry of the wild silkworm. The findings of this study will be helpful for further exploration of insect climbing for foraging and larval mimicry behaviors.

## Data Availability Statement

The datasets presented in this study can be found in online repositories. The names of the repository/repositories and accession number(s) can be found below: https://www.ncbi.nlm.nih.gov/, PRJNA607658.

## Author Contributions

HX conceived and supervised the study. MW drafted the manuscript. YL and MW performed the experiments and generated the mapping population construction, sample collection, BSA analyses, and selection sweep analyses. YC, YL, and SZ collected and analyzed the transcriptome data of the different tissues of the silkworm. HX, WY, and QF revised the manuscript. All authors have read and approved the manuscript.

## Conflict of Interest

The authors declare that the research was conducted in the absence of any commercial or financial relationships that could be construed as a potential conflict of interest.

## Footnotes

1 http://sgp.dna.affrc.go.jp/ComprehensiveGeneSet/

2 https://blast.ncbi.nlm.nih.gov/Blast.cgi

3 http://gigadb.org/dataset/100382

4 https://www.ncbi.nlm.nih.gov/sra/

5 https://www.omicshare.com/

## Supplementary Material

Supplementary Figure 1 Comparisons of index in climbing and non-climbing bulks of white body. **(A)** SNP/InDel index in non-climbing bulk. **(B)** SNP/InDel index in climbing bulk. **(C)** ΔSNP/InDel index between climbing and non-climbing bulks. The candidate region related to climbing are marked with red arrows.

Supplementary Figure 2 Comparisons of index in climbing and non-climbing bulks of dark body. **(A)** SNP/InDel index in non-climbing bulk. **(B)** SNP/InDel index in climbing bulk. **(C)** ΔSNP/InDel index between climbing and non-climbing bulks. The candidate region related to climbing are marked with red arrows.

Supplementary Figure 3 SNP analysis of the candidate domestication genes related to climbing. Allele frequencies in the genetic region of the other three genes. Green blocks indicate the geno mic structure of corresponding genes. The non-synonymous replacement between the domestic silkworm group and the wild silkworm are marked with red arrows.

Supplementary Figure 4 Phylogenetic analysis of ASNA1 in insects. The protein sequence of ASNA1 from silkworm was used as query to blast against all the insect genomes in NCBI database with default settings. Protein sequences with at least 80% identity of ASNA1 in silkworm were downloaded and aligned with ClustalW in MEGA7. The minimum-evolution tree was constructed to show the evolution of ASNA1 in all the collected insect genomes. The ASNA1 in silkworm was marked with black filled circle, and species with two copies of ASNA1 were marked with black filled triangles.

Supplementary Figure 5 Protein alignments of the candidate mutation of ASNA1 in insects. The candidate mutation was divided into two groups (highlighted with cyan and yellow colors, respectively).

Supplementary Figure 6 Protein alignments of the candidate mutations of FAN1 and Pigo in silkworm and five Lepidoptera species. **(A)** Two candidate non-synonymous mutations on FAN1. **(B)** Two candidate non-synonymous mutations on Pigo.

Supplementary Figure 7 Comparisons of index in mimicry and non-mimicry bulks. **(A)** SNP/InDel index in non-mimicry bulk. **(B)** SNP/InDel index in mimicry bulk. **(C)** ΔSNP/InDel index between mimicry and non-mimicry bulks. The candidate region related to climbing are marked with red arrows.

Supplementary Figure 8 Allele frequencies of candidate genes related to mimicry response. **(A)** *Bmor_03579*. **(B)** *Bmor_03547*. **(C)** *Bmor_04035*. **(D)** *Bmor_04019*. **(E)** *Bmor_12045*. **(F)** *Bmor_12007*. **(G)** *Bmor_12006*. **(H)** *Bmor_12005*. **(I)** *Bmor_11996*. **(J)** *Bmor_12350*. **(K)** *Bmor_00240*. Blue, domestic silkworm; black, wild silkworm. The allele frequency in genomic region and flanking 2 kb were shown for each gene.

Supplementary Figure 9 Expression levels of candidate genes related to climbing and mimicry in brains of wild silkworm and domestic silkworm. **(A–D)** Four candidate genes related to climbing ability. **(E–M)** Nine detected candidate genes related to mimicry response. Genes predicted to be positively selected in SGID were marked in red. The FPKM values were normalized to the total reads of corresponding samples and multiplied by 10^7^. Student’s *t*-test was used to estimate the differences between wild silkworm and domestic silkworm. ^∗^*p* < 0.05; ^∗∗^*p* < 0.01.

Supplementary Table 1 Summary of sequence data of parental lines and segregation bulks.

Supplementary Table 2 Annotation of the candidate genes for climbing and mimicry.

Supplementary Data ΔSNP/InDel index calculated in the white and dark body sets used for mapping candidate regions related to climbing ability in silkworm. The average ΔSNP/InDel index was calculated with 1 Mb sliding window with 10 kb step size.

Supplementary Video 1 Climbing and mimicry of BC1. Individuals with strong ability of climbing in BC1. The branches were shaken for observation of the responses of each individual. Individuals response to artificial shaking by stretching their head and thorax to mimic the branch.

Supplementary Video 2 Individuals response to hand touching. Individuals showing strong response to artificial shaking were verified by hand touching.

Supplementary Video 3 Non-mimicry traits of BC1. BC1 individual’s loss of mimicry showed no response and kept eating when touched by hand.
