# Supplementary material for: Genetic Mapping of Climbing and Mimicry: Two Behavioral Traits Degraded During Silkworm Domestication
Source: Front Genet. 2020 Dec 17;11:566961. doi: 10.3389/fgene.2020.566961 (PMC7773896; doi:10.3389/fgene.2020.566961)
Supplement: Supplementary Figure 1 — Comparisons of index in climbing and non-climbing bulks of white body. (A) SNP/InDel index in non-climbing bulk. (B) SNP/InDel index in climbing bulk. (C) ΔSNP/InDel index between climbing and non-climbing bulks. The candidate region related to climbing are marked with red arrows. [file Data_Sheet_1.docx]

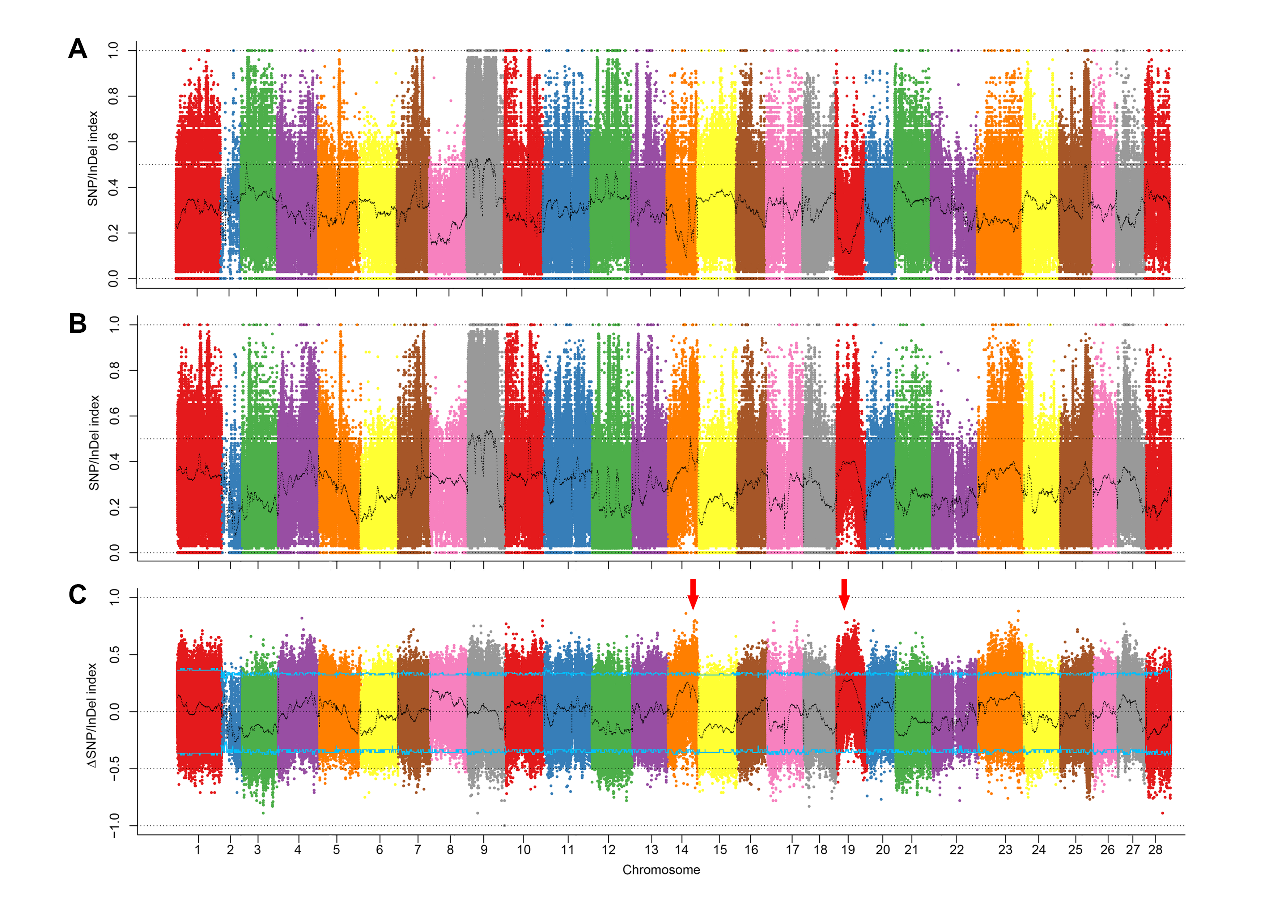


**Figure S1. Comparisons of index in climbing and non-climbing bulks of white body. (A)** SNP/InDel index in non-climbing bulk. **(B)** SNP/InDel index in climbing bulk. **(C)**ΔSNP/InDel index between climbing and non-climbing bulks. The candidate region related to climbing are marked with red arrows.


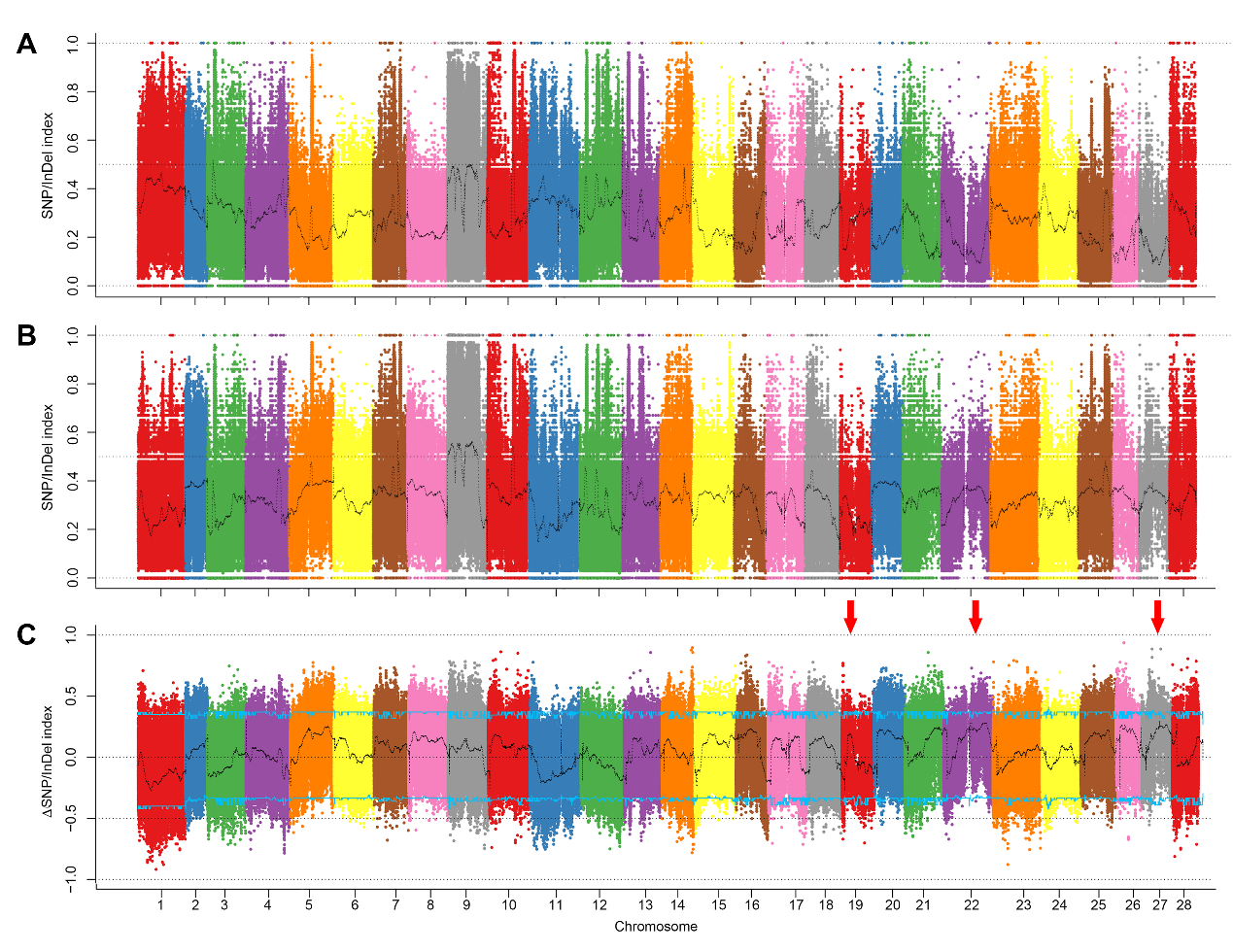


**Figure S2. Comparisons of index in climbing and non-climbing bulks of dark body. (A)** SNP/InDel index in non-climbing bulk. **(B)** SNP/InDel index in climbing bulk. **(C)**ΔSNP/InDel index between climbing and non-climbing bulks. The candidate region related to climbing are marked with red arrows.


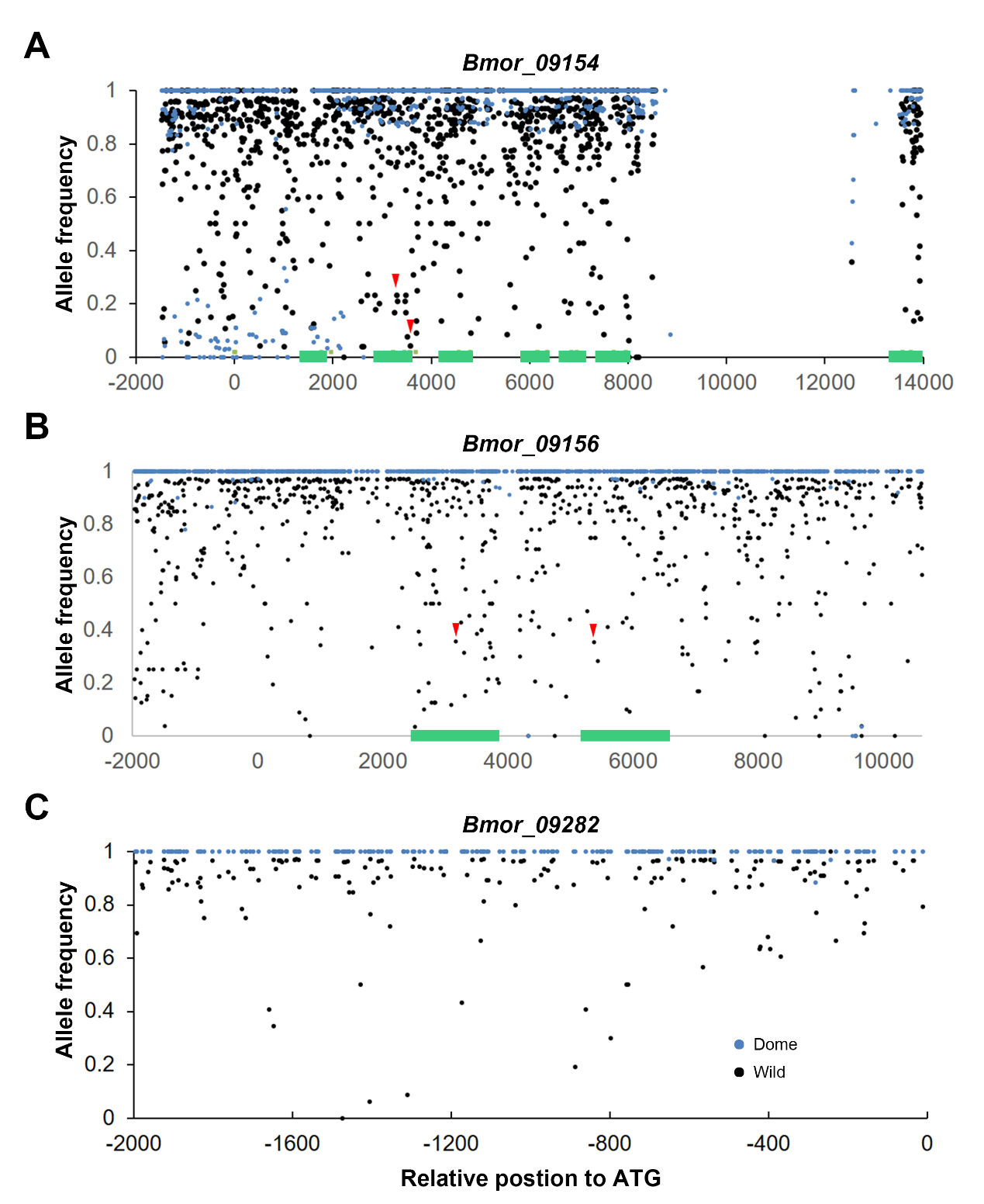


**Figure S3 SNP analysis of the candidate domesticated genes related to climbing loci.** The distributions of Allele frequency of the other three candidate domesticated genes were shown. Green blocks indicate the genomic structure of the genes. Red arrows indicate the non-synonymous replacement in the domestic silkworm group compared with the wild silkworm.Dome, domestic silkworm; Wild, wild silkworm.

**
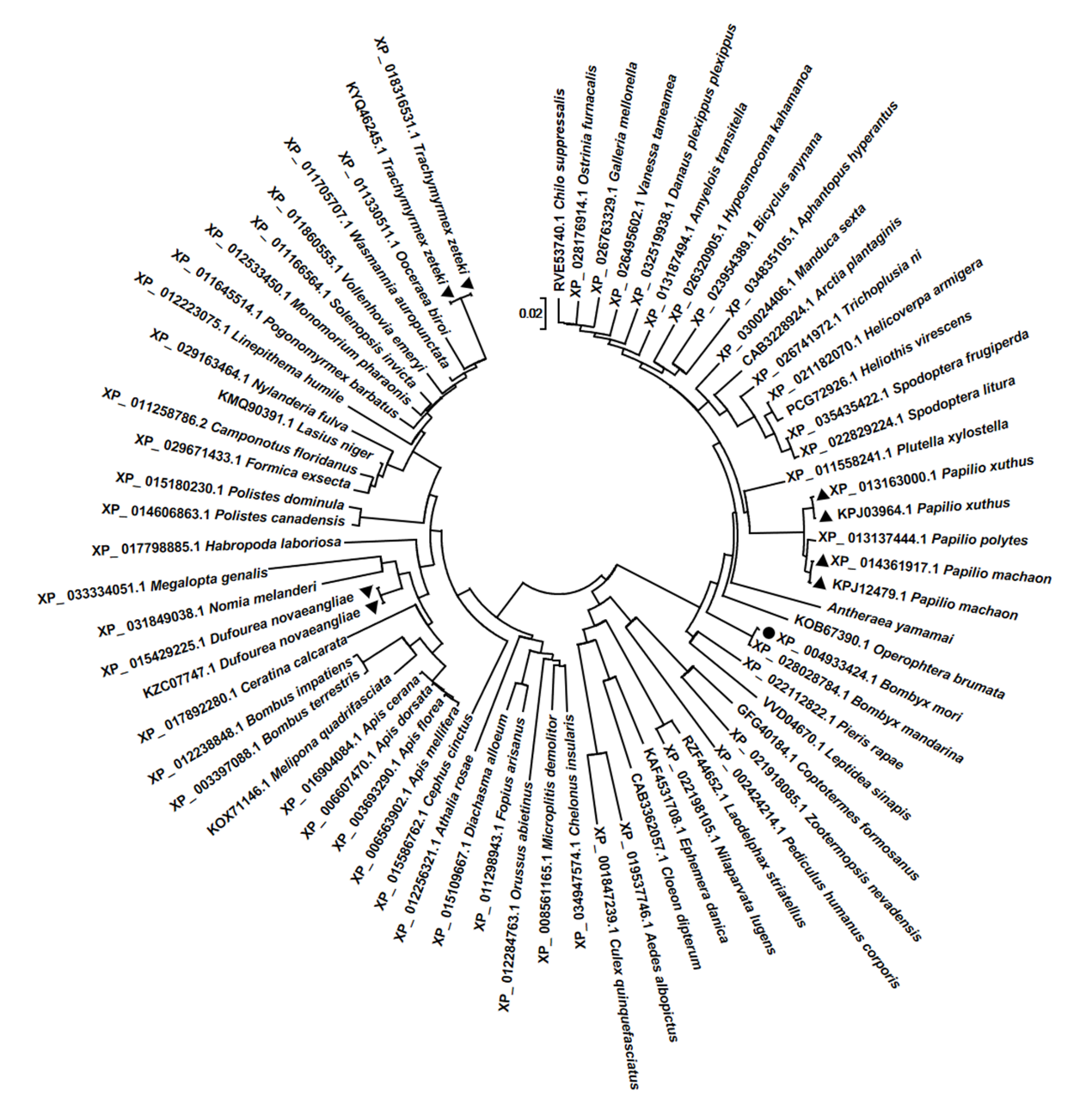
**

**Figure S4Phylogenetic analysis of ASNA1 in insects.**The protein sequence of ASNA1 from silkworm was used as query to blast against all the insect genomes in NCBIdatabase with default settings. Protein sequences with at least 80% identity of ASNA1 in silkworm were downloaded and aligned with ClustalW in MEGA7. The minimum-evolution tree was constructed to show the evolution of ASNA1 in all the collected insect genomes. The ASNA1 in silkworm was marked with black filled circle, and species with two copies of ASNA1 were marked with black filled triangles.


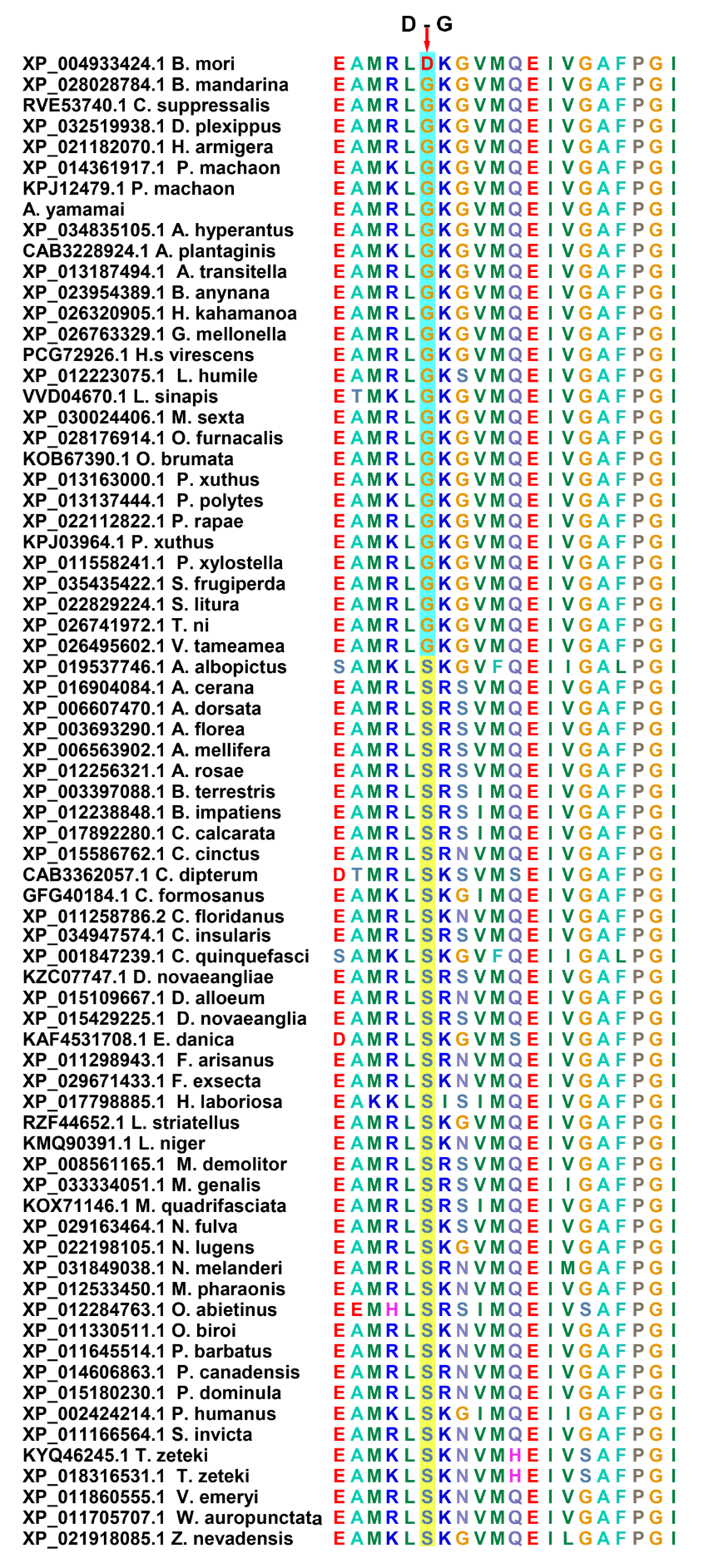


**Figure S5Protein alignments of the candidate mutation of ASNA1 in insects.**The candidate mutation was divided into two groups (highlighted with cyan and yellow colors, respectively).


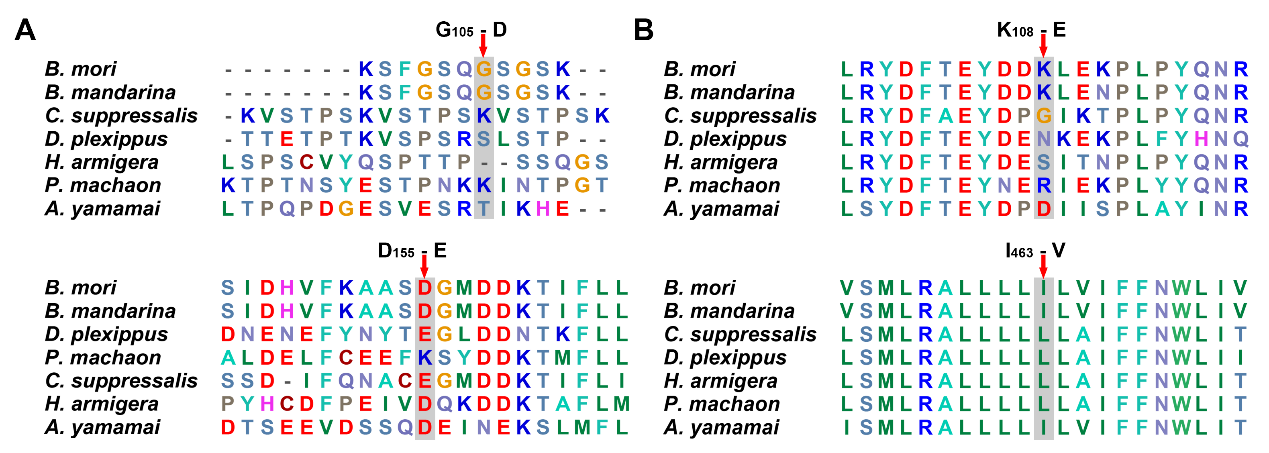


**Figure S6Protein alignments of the candidate mutations of FAN-1 andPigo in silkworm and five Lepidoptera species.**(A) Two candidate non-synonymous mutations on FAN-1. (B) Two candidate non-synonymous mutations on Pigo.


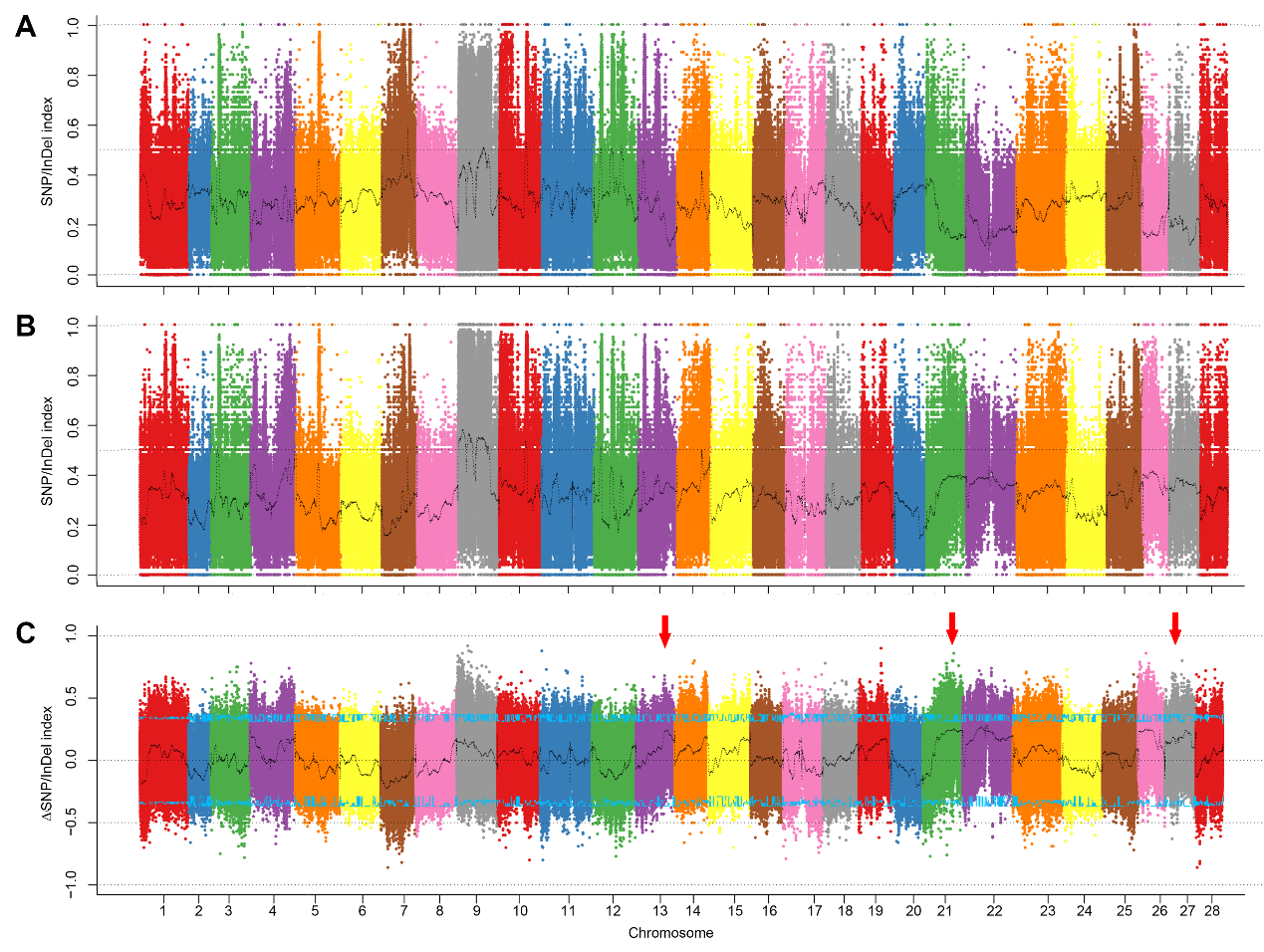
**Figure S7. Comparisons of index in mimicry and non-mimicry bulks. (A)** SNP/InDel index in non-mimicry bulk. **(B)** SNP/InDel index in mimicry bulk. **(C)** ΔSNP/InDel index between mimicry and non-mimicry bulks. The candidate region related to climbing are marked with red arrows.


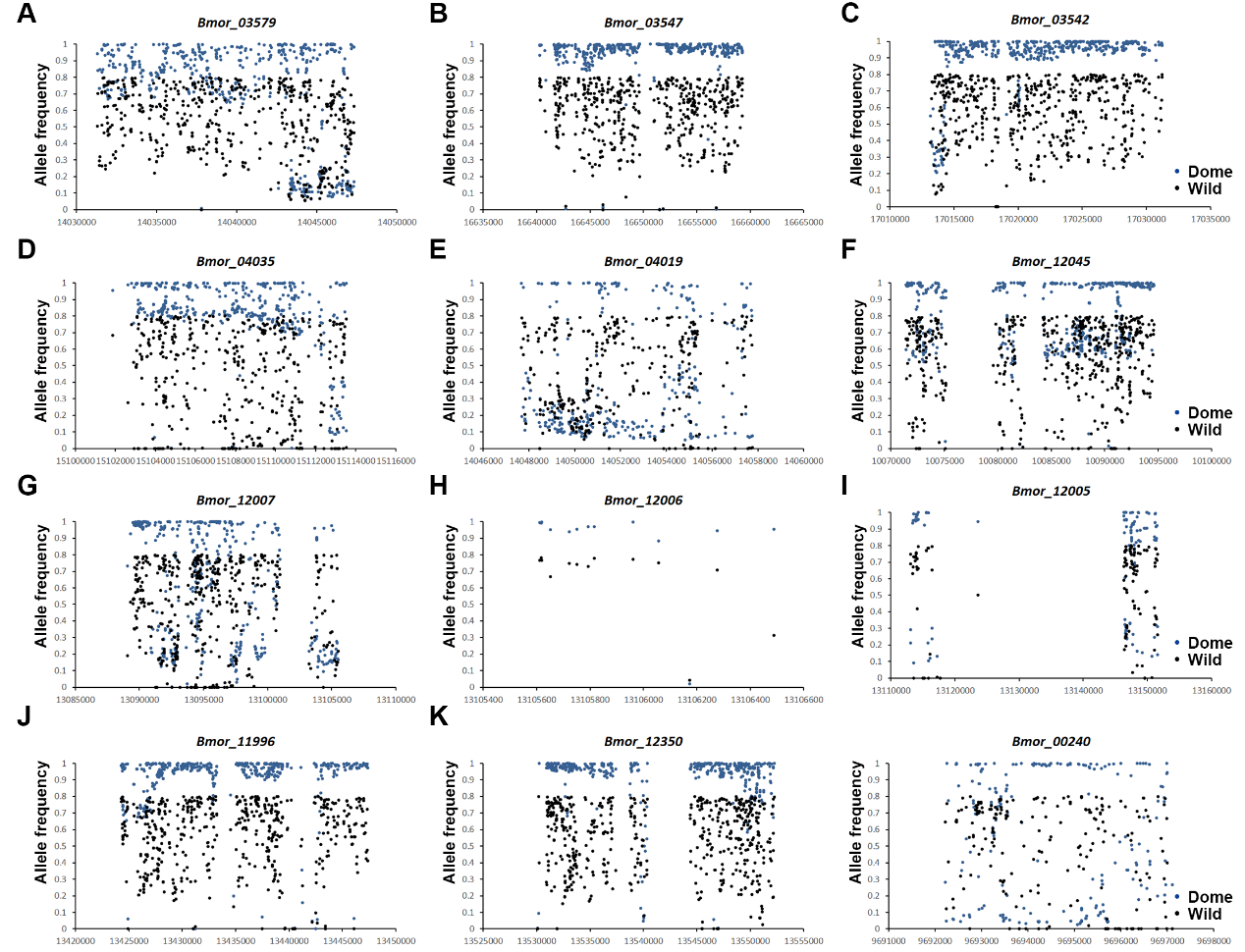


**Figure S8Allele frequencies of candidate genes related to mimicry response.**(A) *Bmor_03579*. (B) *Bmor_03547*. (C) *Bmor_04035*. (D) *Bmor_04019*. (E) *Bmor_12045*. (F) *Bmor_12007*. (G) *Bmor_12006*. (H) *Bmor_12005*. (I) *Bmor_11996*. (J) *Bmor_12350*. (K) *Bmor_00240*. Blue, domestic silkworm; black, wild silkworm. The Allele frequency in genomic region and flanking 2 kb were shown for each gene.


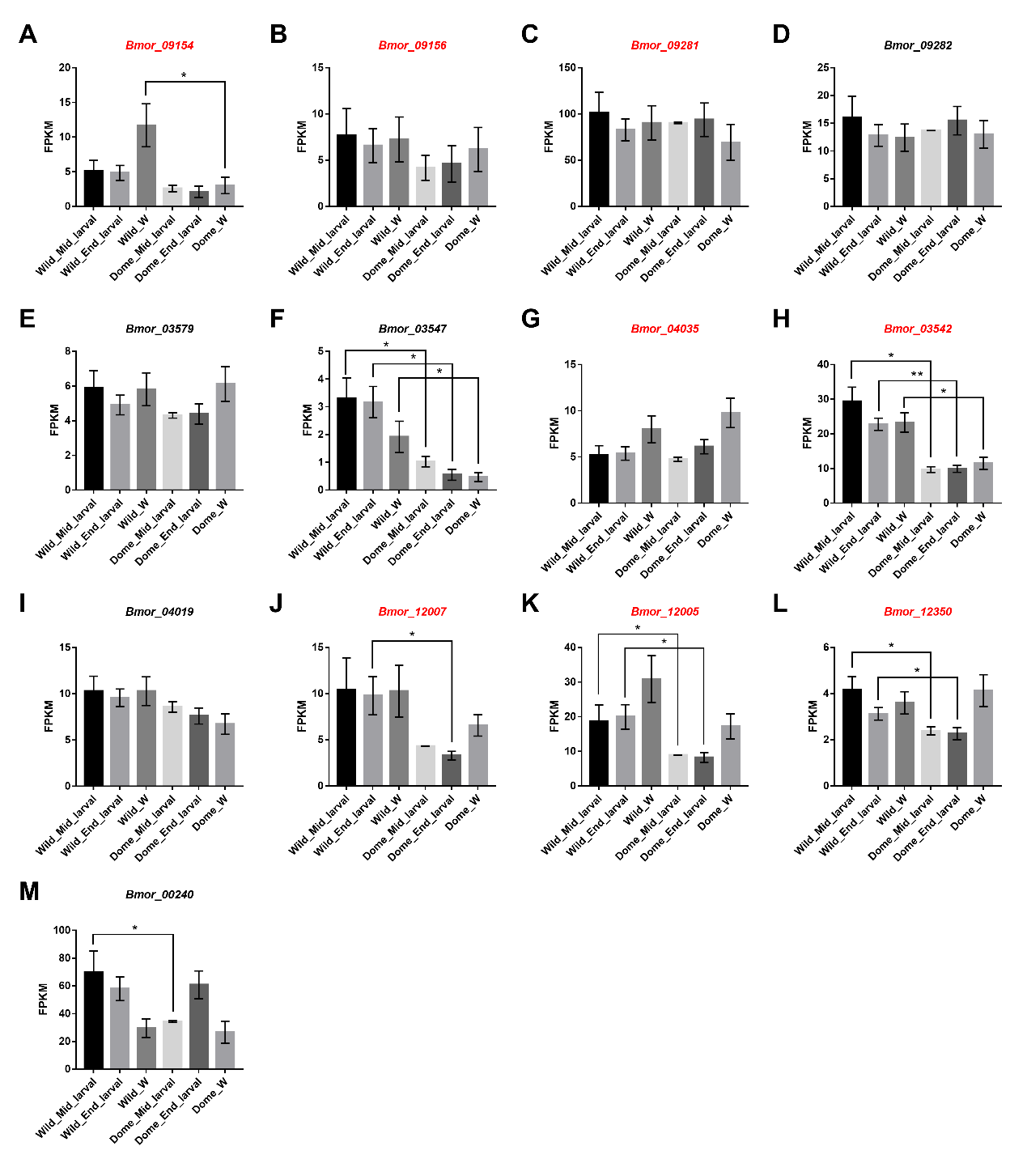


**Figure S9Expression levels of candidate genes related to climbing and mimicry in brains of wild silkworm and domestic silkworm.** (A-D) Four candidate genes related to climbing ability. (E-M) Nine detected candidate genes related to mimicry response. Genes predicted to be positively selected in SGID were marked in red. The FPKM values were normalized to the total reads of corresponding samples and multiplied by 10^7^. Student’s *t*-test was used to estimate the differences between wild silkworm and domestic silkworm. *, *p*-value <0.05; **, *p*-value <0.01.

**Table S1. Summary of sequence data for parental lines and bulked segregationpools**

| **Samples** | **# samples for bulked-seq** | **Clean data (bp)** | **Average depth**  **(X)** | **Mapping rate (%)** |
| --- | --- | --- | --- | --- |
| P_P50 | / | 10,044,350,400 | 21.08 | 95.18 |
| P_Wild | / | 10,924,272,600 | 20.39 | 90.22 |
| White_Climbing | 20 | 19,567,557,300 | 33.98 | 93.89 |
| White_Non-Climbing | 20 | 16,444,811,700 | 30.55 | 93.67 |
| Dark_Climbing | 20 | 15,817,231,200 | 28.81 | 94.01 |
| Dark_Non-Climbing | 20 | 16,804,396,800 | 34.01 | 93.34 |
| Mimicry | 18 | 17,502,161,400 | 32.68 | 93.91 |
| Non-Mimicry | 20 | 18,683,210,100 | 37.11 | 94.07 |

**Table S2.Annotation of candidate genes for climbing and mimicry trait loci.**The gene IDs from SilkDB and SGID were obtained by protein blast. The *Fst*, *π*and selection types were imported from SGID.

| **GeneID** | **SilkDB ID** | **SGID** | ***Fst*** | **π (Dome/Wild)** | **Selection type** | **Chr** | | **Putative function** |
| --- | --- | --- | --- | --- | --- | --- | --- | --- |
| **Climbing related** | | | | | | | | |
| *Bmor_09154* | BMSK0011054 | KWMTBOMO11407 | 0.3907 | 230.1063/350.5464 | P | 19 | fanconi-associated nuclease 1-like | |
| *Bmor_09156* | BMSK0011061 | KWMTBOMO11415 | 0.3790 | 218.4441/515.6968 | P | 19 | GPI ethanolamine phosphate transferase 3 | |
| *Bmor_09281* | BMSK0011060 | KWMTBOMO11416 | 0.3627 | 223.8520/550.3018 | P | 19 | ATPase ASNA1 homolog | |
| *Bmor_09282* | BMSK0011059 | KWMTBOMO11414 | 0.4108 | 203.4659/512.6542 | Uncertain | 19 | uncharacterized protein LOC105842589 | |
| **Mimic related** | | | | | | | | |
| *Bmor_03579* | BMSK0007483 | KWMTBOMO07708 | 0.2439 | 36.3214/201.9892 | Uncertain | 13 | TBC domain-containing protein kinase-like protein | |
| *Bmor_03547* | BMSK0007420 | KWMTBOMO07630 | 0.3234 | 221.7215/402.0890 | Uncertain | 13 | glycine receptor subunit alpha-4 | |
| *Bmor_04035* | BMSK0007456 | KWMTBOMO07666 | 0.3838 | 260.5837/578.2070 | P | 13 | uncharacterized protein LOC101743788 | |
| *Bmor_03542* | BMSK0012061 | KWMTBOMO07620 | 0.4107 | 203.2076/266.5317 | P | 13 | periodic tryptophan protein 2 homolog | |
| *Bmor_04019* | BMSK0007482 | KWMTBOMO07707 | 0.3201 | 201.1713/453.0107 | Uncertain | 13 | putative leucine-rich repeat-containing protein | |
| *Bmor_12045* | BMSK0010951 | KWMTBOMO12573 | 0.3289 | 267.7901/270.7584 | Uncertain | 21 | neuropeptide receptor A28 | |
| *Bmor_12007* | BMSK0012319 | KWMTBOMO12724 | 0.2409 | 18.8940/29.3783 | P | 21 | A-kinase anchor protein 17A | |
| *Bmor_12006* | BMSK0012320 | KWMTBOMO12724 | 0.2409 | 18.8940/29.3783 | P | 21 | A-kinase anchor protein 17A | |
| *Bmor_12005* | BMSK0012322 | KWMTBOMO12725 | 0.2364 | 2.0548/20.2120 | P | 21 | uncharacterized protein LOC101741859 | |
| *Bmor_11996* | BMSK0012347 | KWMTBOMO12750 | 0.5677 | 1.0015/22.1247 | Uncertain | 21 | neuropeptide receptor A29 | |
| *Bmor_12350* | BMSK0012350 | KWMTBOMO12752 | 0.5513 | 2.8323/26.4860 | P | 21 | AP-3 complex subunit beta-2 | |
| *Bmor_00240* | BMSK0015729 | KWMTBOMO16139 | 0.3411 | 214.3256/237.4503 | Uncertain | 27 | facilitated trehalose transporter Tret1-like | |
